# Supplementary material for: Integrating Fly Ash-Controlled Surface Morphology and Candle Grease Coating: Access to Highly Hydrophobic Poly (L-lactic Acid) Composite for Anti-Icing Application
Source: Nanomaterials (Basel). 2023 Mar 30;13(7):1230. doi: 10.3390/nano13071230 (PMC10096872; doi:10.3390/nano13071230)
Supplement: Supplementary file 1 [file nanomaterials-13-01230-s001.zip › nanomaterials-2283246-supplementary.pdf]

Supplementary Materials for

Integrating Fly Ash-Controlled Surface Morphology and Candle Grease Coating: Access to Highly Hydrophobic Poly (L-Lactic Acid) Composite for Anti-Icing Application

Zhiqiang Jiang [1,2], Bai Xue [1,2,3,*], Xiaoping Mai [1,2], Changmei Wu [1,2], Lingjun Zeng [1,2], Lan Xie [1,2,3,*] and Qiang Zheng [4]

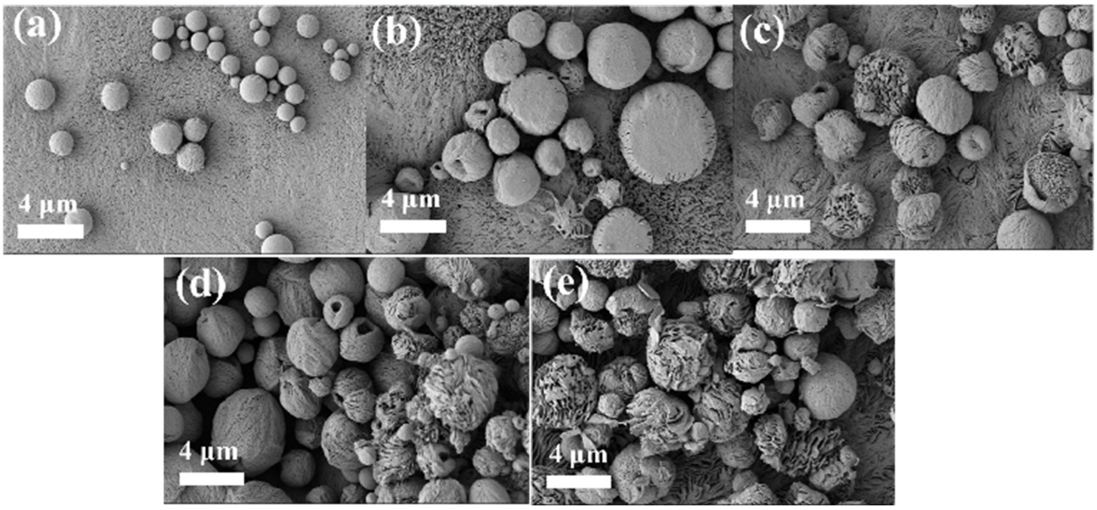

Figure S1. Surface SEM images of (**a**) pure PLLA, (**b**) 1 wt% PLLA/FA@KH570, (**c**) 3 wt% PLLA/FA@KH570, (**d**) 5 wt% PLLA/FA@KH570, and (**e**) 7 wt% PLLA/FA@KH570 composites.

Static contact angle testing was performed by contact angle measuring instrument and corresponding analysis software CAST3.0. The contact angle measuring instrument was equipped with a platform, needle tube and microscope. Place the test surface of the sample on a movable platform and dropwise add droplets from the needle tubing onto the surface of the composites. The image was developed by the analysis software and the contact angle was measured. The average contact angle was obtained by measuring at any five points on the surface of the composites.

In addition, we know that, according to the adhesion theory, rose petal-like surfaces were not only hydrophobic but also somewhat adherent. As could be seen from Figure 4, the surface of the 5 wt% PLLA/FA@KH570 composite had a petal-like structure. Correspondingly, we conducted a rolling angle test and the results were as expected.

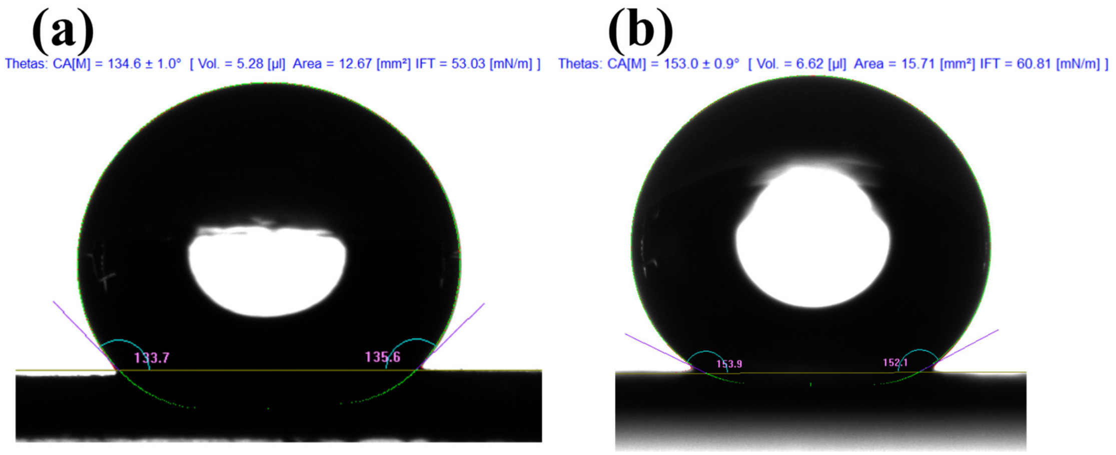

Figure S2. Static contact angles of pure PLLA (**a**) and 5 wt% PLLA/FA@KH570 composite (**b**), respectively.

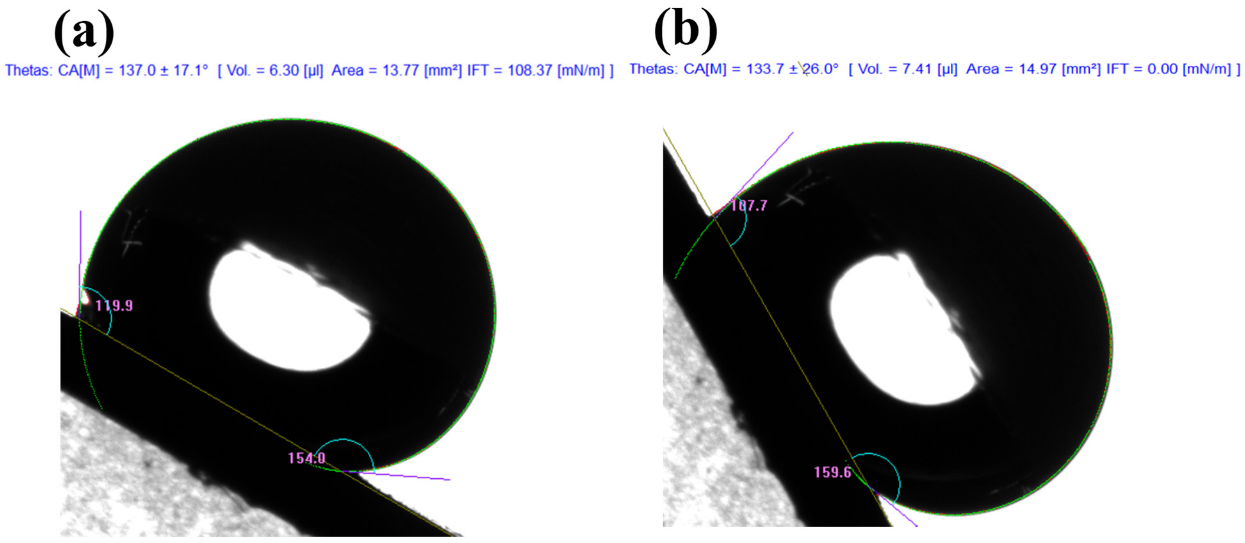

Figure S3. Rolling angle photos of pure PLLA at the tilt angle of 30° (**a**) and 60° (**b**), respectively.

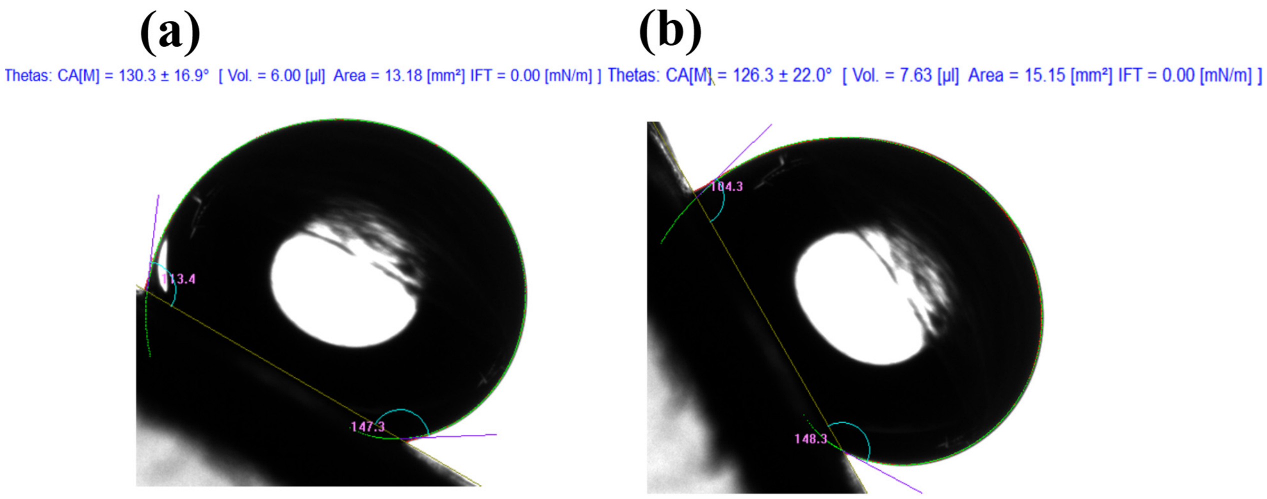

Figure S4. Rolling angle photos of 1 wt% PLLA/FA@KH570 composite at the tilt angle of 30° (**a**) and 60° (**b**), respectively.

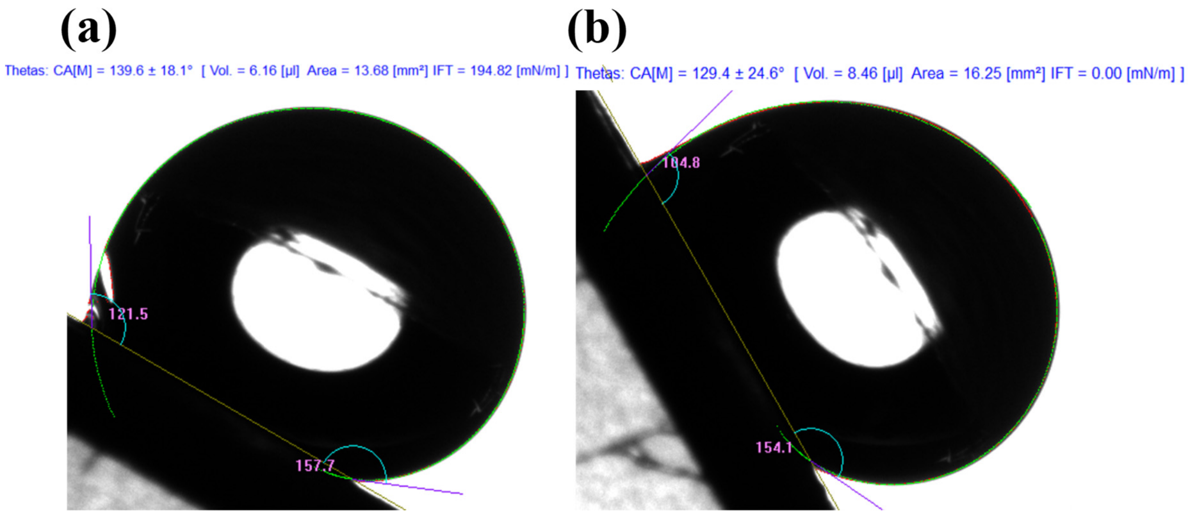

Figure S5. Rolling angle photos of 3 wt% PLLA/FA@KH570 composite at the tilt angle of 30° (**a**) and 60° (**b**), respectively.

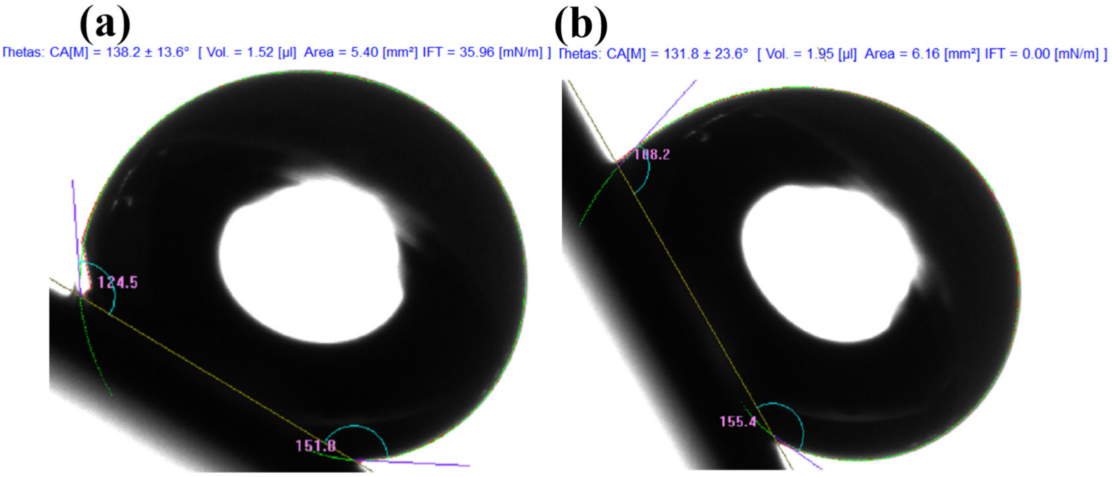

Figure S6. Rolling angle photos of 5 wt% PLLA/FA@KH570 composite at the tilt angle of 30° (**a**) and 60° (**b**), respectively.

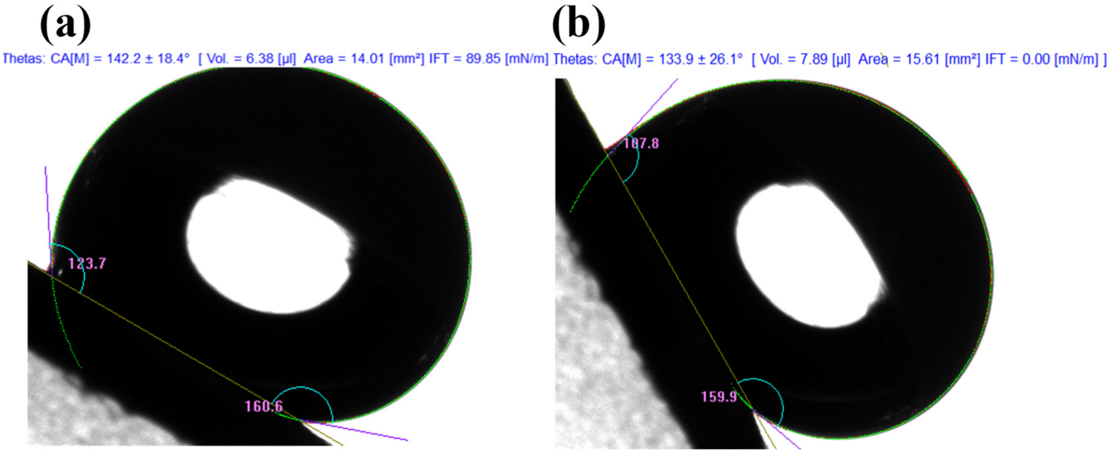

Figure S7. Rolling angle photos of 7 wt% PLLA/FA@KH570 composite at the tilt angle of 30° (**a**) and 60° (**b**), respectively.

Table S1. XRD data of FA.

Component	2θ	hkl
SiO_2	18.2°	(312)
SiO_2	26.7°	(513)
Al_2O_3	35.4°	(311)
Fe_2O_3	33.3°	(311)
$3Al_2O_3·2SiO_2$	40.1°	(210)

Table S2. Data analysis tables for pure PLLA and 5 wt% PLLA/FA@KH570 composite.

Sample Code	Glass Transition Temperature (°C)	Melting Temperature (°C)	Degree of Crystallinity (%)	Contact Angles (°)	Weight Increase (%)	Delay Time (s)
pure PLLA	55.9	167.3	55.1	133	13.4	159
5 wt% PLLA/FA@KH570	58.9	166.7	78.2	151	5.3	410
